# Hypothermia effects on neuronal plasticity post spinal cord injury

**DOI:** 10.1371/journal.pone.0301430

**Published:** 2024-04-05

**Authors:** Hasan Al-Nashash, Ka-Leung Wong, Angelo H. ALL

**Affiliations:** 1 Department of Electrical Engineering, College of Engineering, American University of Sharjah, Sharjah, United Arab Emirates; 2 Department of Applied Biology and Chemical Technology, The Hong Kong Polytechnic University, Hong Kong, China; 3 Department of Chemistry, Faculty of Science, Hong Kong Baptist University, Hong Kong, China; Belgrade University Faculty of Medicine, SERBIA

## Abstract

**Background:**

SCI is a time-sensitive debilitating neurological condition without treatment options. Although the central nervous system is not programmed for effective endogenous repairs or regeneration, neuroplasticity partially compensates for the dysfunction consequences of SCI.

**Objective and hypothesis:**

The purpose of our study is to investigate whether early induction of hypothermia impacts neuronal tissue compensatory mechanisms. Our hypothesis is that although neuroplasticity happens within the neuropathways, both above (forelimbs) and below (hindlimbs) the site of spinal cord injury (SCI), hypothermia further influences the upper limbs’ SSEP signals, even when the SCI is mid-thoracic.

**Study design:**

A total of 30 male and female adult rats are randomly assigned to four groups (n = 7): sham group, control group undergoing only laminectomy, injury group with normothermia (37°C), and injury group with hypothermia (32°C +/-0.5°C).

**Methods:**

The NYU-Impactor is used to induce mid-thoracic (T8) moderate (12.5 mm) midline contusive injury in rats. Somatosensory evoked potential (SSEP) is an objective and non-invasive procedure to assess the functionality of selective neuropathways. SSEP monitoring of baseline, and on days 4 and 7 post-SCI are performed.

**Results:**

Statistical analysis shows that there are significant differences between the SSEP signal amplitudes recorded when stimulating either forelimb in the group of rats with normothermia compared to the rats treated with 2h of hypothermia on day 4 (left forelimb, *p = 0*.*0417* and right forelimb, *p = 0*.*0012*) and on day 7 (left forelimb, *p = 0*.*0332* and right forelimb, *p = 0*.*0133*) post-SCI.

**Conclusion:**

Our results show that the forelimbs SSEP signals from the two groups of injuries with and without hypothermia have statistically significant differences on days 4 and 7. This indicates the neuroprotective effect of early hypothermia and its influences on stimulating further the neuroplasticity within the upper limbs neural network post-SCI. Timely detection of neuroplasticity and identifying the endogenous and exogenous factors have clinical applications in planning a more effective rehabilitation and functional electrical stimulation (FES) interventions in SCI patients.

## Introduction

Few therapeutic options exist for patients suffering from spinal cord injury (SCI). In general, the injury could be categorized into five types: contusion, compression, focal demyelination, transection, and ischemic. Each type of SCI has unique pathophysiological features of onset and progress, and the research models of SCI are based on these distinctive characteristics. For instance: during sudden impact or contusive injury, the energy of impact is absorbed mainly by the larger caliber neurons, causing their disruption. However, smaller caliber neurons are more vulnerable to a compression injury, which has less impact force but exists for much longer time, similar to the growth of tumors. On the other hand, in the case of focal demyelination (Multiple Sclerosis like pathologies), the myelin membrane, not neurons, is the leading pathophysiology site. The transection injuries (such as penetrating wounds) induce damage to all neural cells indiscriminately. Since the spinal cord is extremely vulnerable, even small interruption of blood flow (blockage, hemorrhage, or major surgical procedures) may initiate the cascade of ischemic injury [1–6].

SCI is a time-sensitive debilitating neurological condition with progressive irregular neurodegeneration. Contusion injuries are mainly caused by falls, vehicles, or sportive accidents, in which high-impact energy is transferred to a small area of the spinal cord parenchyma suddenly. Clinically, contusive SCI is the most prevalent type of injury among young male adults [7]. Initially, SCI is characterized by ischemia injury, followed by oxidative stress and the cascade of devastating inflammatory events that will cause apoptosis and necrosis of the neuronal cells locally and in the surrounding parenchyma. Depending on the severity and location of the primary injury, the results often are significant motor, sensory, and autonomic dysfunctions causing paralysis in body, arms, and legs (tetraplegia, quadriplegia) [8, 9]. Even though the survival rate from SCI is significantly higher than the past, there are still no efficacious treatments. Most therapeutic interventions are designed to either stop or slow the progress of injury or are palliative to relieve symptoms. Unfortunately, therapeutic options are even less effective in patients suffering from the chronic form of SCI [10, 11].

SCIs are classified as acute (the first few hours), sub-acute (the first few days), and chronic (beyond a week or so), though there is no exact time definition or distinctive line that separates one phase from the other. The acute phase of SCI has consistently been the focus of designing, administrating, and managing most therapeutic interventions hereinto. It has been reported that although inducing hypothermia per se’ would not be an exact therapeutic approach because of its temporary effect on slowing the pathophysiological events and not treating the cause of injury, it could undoubtedly be considered an effective neuroprotective strategy during the acute and sub-acute phases of SCI. Hypothermia, when administered early post-contusive SCI, would provide physicians with a longer therapeutic time-window to manage treatments that otherwise would be ineffective during the very inflammatory acute phase [4, 12, 13]. For instance, the potential benefits of stem cell replacement therapy such as human oligodendrocyte progenitor cells have been extensively investigated. Nevertheless, by and large cell therapies would not be much effective during the acute phases of SCI, mainly because most intra-parenchyma transplanted cells would not survive, integrate, proliferate, and become functional due to the host very hostile microenvironment [14–26]. Moreover, cell therapies in SCI would be more efficient, when combined with rehabilitation and functional electrical stimulation [27].

The neuroprotective effect of hypothermia has been extensively investigated by us and others. We have shown both neuro-electrophysiological and behavioral improvements after inducing early hypothermia in various neurotraumas to a great extent. The early neuroplasticity post-neurotrauma has also been reported largely. However, the purpose of our study here is to investigate whether early induction of hypothermia impacts neuronal tissue compensatory mechanisms. Our hypothesis is that although neuroplasticity happens within the neuropathways both above (forelimbs) and below (hindlimbs) the site of spinal cord injury (SCI), hypothermia further influences the forelimbs SSEP signals, even when the SCI is mid-thoracic. Here for the first time, we present the stimulating influence of hypothermia on neuroplasticity of the upper limbs’ neuropathways / network post mid-thoracic SCI.

## Materials and methods

### Animal groups

A total of 30 male and female adult (~250g) Wistar rats were randomly assigned to the following four groups (n = 7 per group): (i) Sham group with no laminectomy and no injury, (ii) Control group undergoing only laminectomy, (iii) Injury group with normothermia at 37°C, and (iv) Injury group with 2 h hypothermia at 32°C +/-0.5°C that started 2h after the injury. Two rats were also excluded due to experimental deviations.

### Anesthesia

For the surgical procedures and induction/maintenance of hypothermia, rats underwent general anesthesia using intraperitoneal injection (ip) of 0.2 ml freshly mixed cocktail of ketamine (50 mg/kg), xylazine (5 mg/kg) and acepromazine (1 mg/kg). However, since the ketamine cocktail anesthesia suppresses the brain SSEP signals, a mixture of 1.5% isoflurane, 85% oxygen, and room air (delivered by the rodent vaporizer and ventilator at the rate of 1.5 L per minute via an anesthesia mask connected to a diaphragm with a C-pram circuit), was used for the neuro-electrophysiology (SSEP) monitoring. We periodically tested rats’ responses to noxious stimuli to ensure that during the entire surgical procedures, hypothermia induction, and electrophysiological monitoring pain and discomfort were avoided [1, 28, 29].

### Laminectomy

It is a safe and easy procedure to expose the dorsal part of the spinal cord. It is performed by making a ~3 cm skin incision and retracting the paravertebral muscles while rats are under deep general anesthesia. Then, the T8 vertebrae is identified, and the lamina at T8 is removed carefully without opening or damaging the dura [1–3].

### Injury

**Fig 1:**
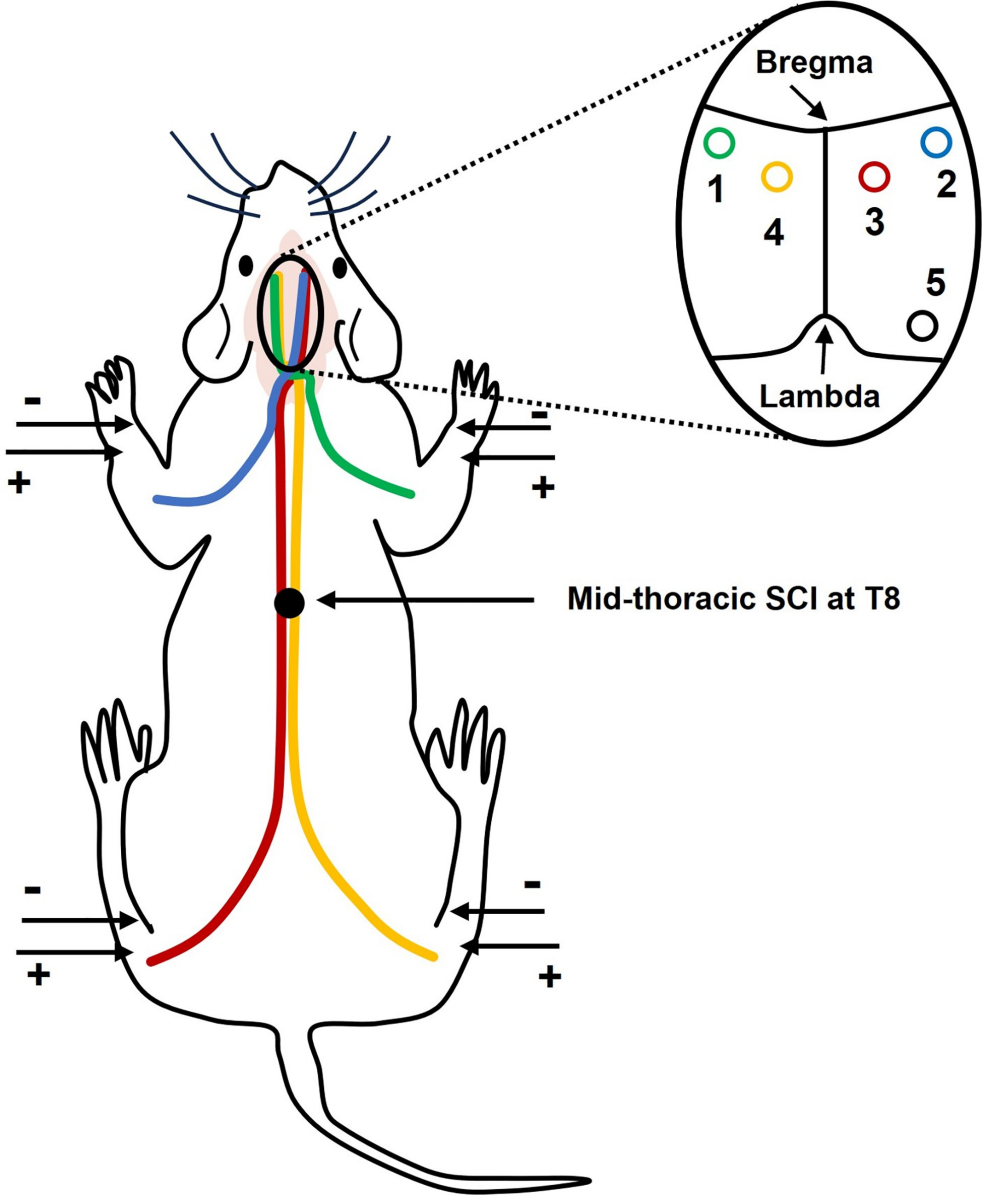
Hindlimbs (red and yellow) and forelimb (green and blue) neuropathways and their corresponding contralateral sensory projections on the right and left cortices are shown. Left and right Median and Tibial nerves on each limb are stimulated via a pair of subcutaneous needle electrodes. The epicenter of SCI is mid-thoracic (T8). Hindlimb neuropathways originate below the injury site, and their propagations are affected by the midline injury. Whereas the forelimb neuropathways are entirely above the injury site.

The laminectomy exposes ~0.5 cm of the dorsal part of the T8 segment, which has ~2–3 mm diameter (Fig 1). This region will be the center for midline contusive injury, using the NYU-MASCIS weight-drop impactor (developed by Prof. Wise Young, Rutgers University) [1]. The NYU-impactor, a well-established contusive model of SCI in rodents, consists of a 10 g rod with an impact surface of 2 mm diameter that is released from a height of 12.5 mm to produce a moderate injury. The device records the impact trajectory, height, time, and velocity of the impact to ensure consistency and reproducibility of injury among all rats. After the injury, the paravertebral muscles will be sutured in layers, and the skin will be sealed [30].

### Animal care

All *in vivo* procedures were approved by the Hong Kong Baptist University Institutional Animal Care and Use Committee (IACUC) and Hong Kong Department of Health {the approval number is: (21–24) in DH/HT&A/8/2/6 Pt.3}. All *in vivo* animal procedures followed the Neuroscience Research and the US-NIH guidelines. We confirm that we followed all the institutional and governmental regulations concerning the ethical and legal issues governing biomedical research.

A rodent heating pad is used to keep rats’ body temperature at 37 +/- 0.5°C during surgical and monitoring procedures. After surgery and injury, saline (10cc/day, intradermal) is given for 2 days, Gentamicin (5 mg/kg, intramuscular) for 5 days, and Buprenex analgesic (0.3 mg/kg, subcutaneous) for 3 days. Their bladder is expressed manually twice a day and until spontaneous voiding is observed. Rats will be under observation for signs of pain and infection for 5 days. Rats have access to food and water, placed on the bottom of the cage, freely [1].

### Inducing hypothermia

Others and our team have extensively reported the risk and benefit of inducing hypothermia as a neuroprotective strategy to treat neurotraumas. The risk/benefit ratio is paramount and must always be evaluated. Practically, general, or local mild (34+/-2°C), moderate (32+/-2°C), and severe (30+/-2°C) hypothermia could be induced for short-term (2h), mid-term (4h), and long-term (6h) either invasively (intravascularly via inserting a vein catheter), non-invasively (reducing body’s temperature), or minimally-invasively (supra-spinal cord heat-exchanger). Nonetheless, the side effects of aggressive (very low temperature for a long time) hypothermia could be devastating and outweigh its potential benefits. For instance, if hypothermia is not induced and controlled properly, it could cause life-threatening complication such as peripheral vasoconstriction, bradycardia, coagulopathy dysfunction, increase the blood pH, insulin resistance, and especially shivering, which is due to alteration of thermoregulatory defense mechanisms. On the other hand, although mild (closer to normothermia) hypothermia would not exhibit many risks, it would not produce significant neuroprotective effects either. Our previous studies demonstrated that moderate short-term minimally invasive local hypothermia would render the desired beneficial effects in the case of moderate mid-thoracic contusive SCI in adult (250 g) rats [4, 12, 13, 31–33].

**Fig 2:**
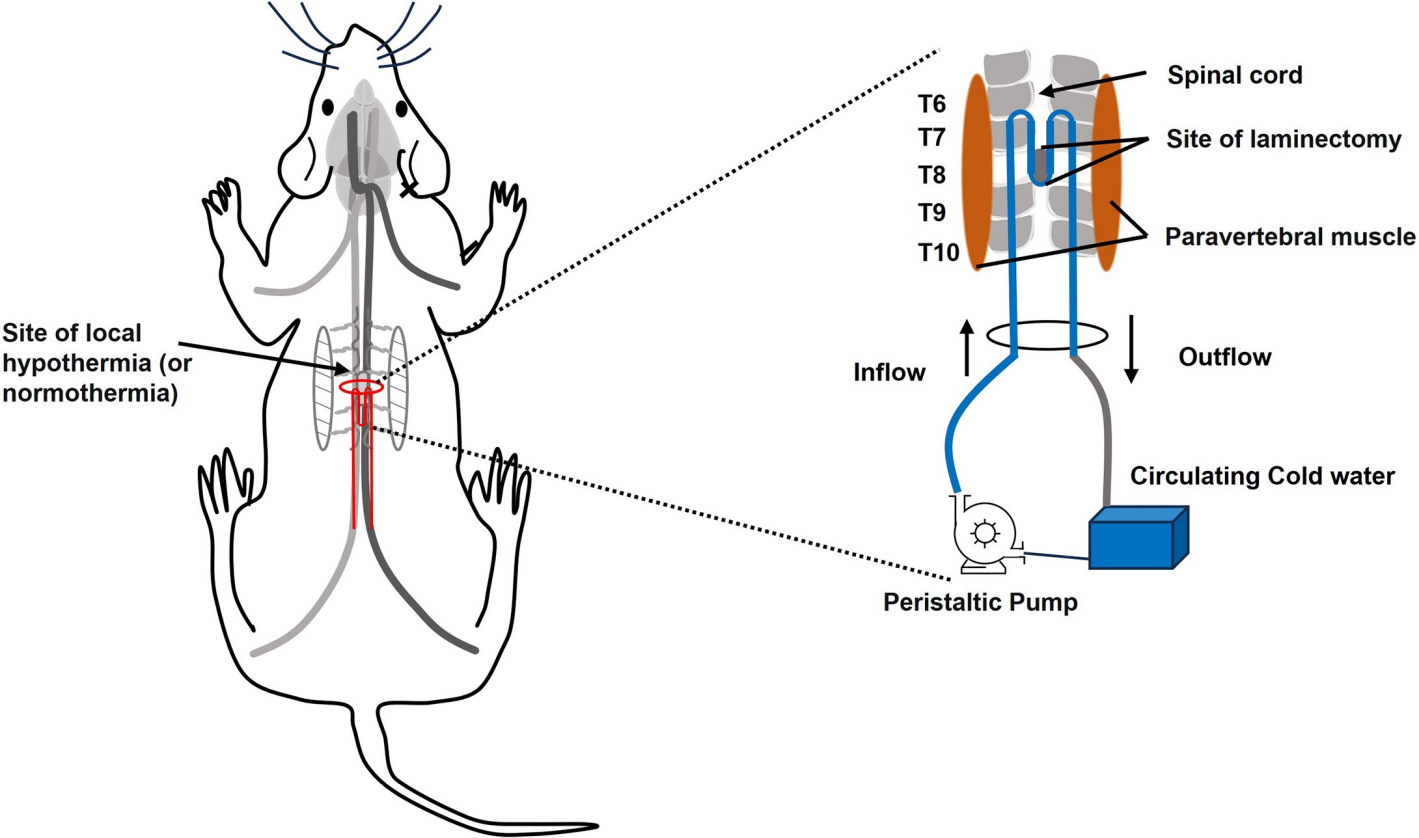
The semi-invasive local hypothermia is delivered to the epicenter of injury (T7-T9) via an M-shaped copper tube connected to the peristaltic pump for circulating cold water around the injury site. By adjusting the circulating time and temperature of cold water, the desired hypothermia degree can easily be achieved and maintained for an extended time.

The semi-invasive local hypothermia is an ideal method to induce and maintain the desired 32°C +/-0.5°C and 37°C degree temperature during the hypothermia and normothermia phases. As published previously, this is done by using a heat-exchanger (a 12 cm length M-shaped 2 mm diameter copper tube connected to a peristaltic pump). After making a ~1 cm transverse skin-only incision and opening a ~10 cm tunnel under the skin and above the vertebrae, simply with inserting the index finger in, the center of the M-shaped tube is placed under the skin and over the T8 segment. Then the two extremities of the M-tube are connected to the peristaltic pump. By adjusting the time and rate of circulating cold water (at 17°C and rate of ~130 mL/min) through the copper tube, the desired local temperature of the spinal cord parenchyma and the surrounding areas is easily achievable (Fig 2) [31–33].

### Monitoring Somatosensory Evoked Potential (SSEP)

Neuroelectrophysiology examinations are the only objective functional assessment of onset, progress, and recovery of neurotraumas. SSEP signals are the cortices responses elicited by external sensory stimuli. They represent the integrity of the entire ascending sensory pathway from the periphery to the brain (Fig 1) [34–37]. Likewise, monitoring Motor Evoked Potential (MEP) enables assessing descending motor pathways via stimulating higher structures of the central nervous system and recording signals from the corresponding muscles or lower motor pathways in the periphery [38–40]. Joint SSEP and MEP monitoring allows quantitative and qualitative assessment of the natural history of injury via monitoring ascending sensory and descending motor pathways [41].

### Skull implantation of recording screw electrode

The SSEP signals are recorded from the corresponding cortices (contralateral to the stimulated limbs) via screw electrodes that are implanted precisely 0.2 mm posterior to bregma and 3.8 mm lateral to midline (corresponding to the cortex forelimbs area) and 2.5 mm posterior to bregma and 2.8 mm lateral to midline (corresponding to the cortex hindlimbs area) on the right and left hemispheres. The fifth reference electrode is implanted on the right frontal bone. Electrodes are carefully implanted just as to make very light and soft contact with the dura mater. Then, they are fixed on the skull with dental cement. This allows for critical long-term longitudinal stable SSEP recording [1].

### Sub-cutaneous stimulating needle electrodes

The SSEP signal is evoked by stimulating a pair of needle electrodes carefully placed near the tibial nerve in the lower limb or median nerve in the upper limb (without contacting the nerve bundle). The pair is connected to an isolated stimulator to deliver positive current pulses of 3.5 mA, 200 microseconds pulse width, and 1 Hz frequency to each limb clockwise with 1 second delay [1].

### Signal analysis

SSEP monitoring, which has also been extensively employed in the clinical setting, is a safe, efficient, reliable, reproducible, and cost-effective method to assess the peripheral and central nervous system. Typically, recordings are followed by real-time and or offline signal processing, which enables scientists to (i) detect the onset and progress of trauma, (ii) qualify and quantify the severity of SCI, and (iii) identify and distinguish even minuscule changes that could be due to the endogenous repair or exogenous therapeutic recovery. A few main methods of SSEP signal processing techniques are shape analysis [42, 43], spectral coherence [44], slope analysis [45], adaptive coherence [46], chirp modeling [47], sparse modeling [48], as well as several other methods [49–53].

### SSEP signal acquisition

To record the SSEP signals, high input impedance and high gain differential amplifiers are used, which is followed by analog-to-digital conversion at a 5 kHz sampling rate. Differential amplifiers are crucial for eliminating common mode interferences, such as 50, 60, or 120 Hz frequencies, as well as low-frequency artifacts caused by electrode-electrolyte interactions. The pulse generator signals, responsible for the stimulation, are recorded simultaneously with the evoked potential signals and saved for the signal processing in a data storage system. Extensive research has demonstrated a proportional correlation between the magnitude changes of SSEP signals and the severity of injury [4].

### SSEP signal processing

The SSEP signals contralateral to the side of limb stimulation are utilized for analysis. To enhance the signal-to-noise ratio, ensemble averaging of 100 to 700 sweeps is employed. Then, peak detection is applied to locate the N1 and P2 peaks of the averaged SSEP signals. Following peak detection, N1-P2 amplitude of the SSEP signals is measured. All signal processing procedures are executed using MATLAB R2022a, developed by The MathWorks, Inc. [48, 50, 52].

### SSEP signal presentation

Normalization of the SSEP averaged signals is performed relative to the respective baseline signal. The term "relative" indicates that the amplitude of the SSEP is measured in relation to the amplitude of the corresponding baseline SSEP signal. This is achieved by dividing the N1-P2 peak-to-peak amplitude of SSEP signals by the N1-P2 peak-to-peak amplitude of the corresponding baseline. Noting that since the mean relative SSEP amplitudes corresponding to forelimb stimulation with normothermia was found to be 2, in favor of reducing the effect of outliers, the extreme values higher than 4 (which is double the mean relative amplitude) were trimmed to 4. Similarly, for the hypothermia data, the mean relative SSEP amplitudes was found to be 3 and therefore, the outliners values higher than 6 (double the mean value) were trimmed to 6.

### Statistical analysis

Various pairwise multivariate statistical tests with 95% confidence level are conducted on relative SSEP amplitudes. In the laminectomy group, t-test analysis is performed between prior (baseline) and post laminectomy. In the injury groups, t-test analysis is performed between the baseline and day 4 and baseline and day 7. The null hypothesis tested is that the mean of relative SSEP amplitudes remains the same on different days both before and after laminectomy, injury and with and without hypothermia. In addition, ANOVA test was performed on the data recorded from normothermia and hypothermia. The null hypothesis was that the mean SSEP relative amplitude is the same for normothermia and hypothermia before and after injury.

**Fig 3:**
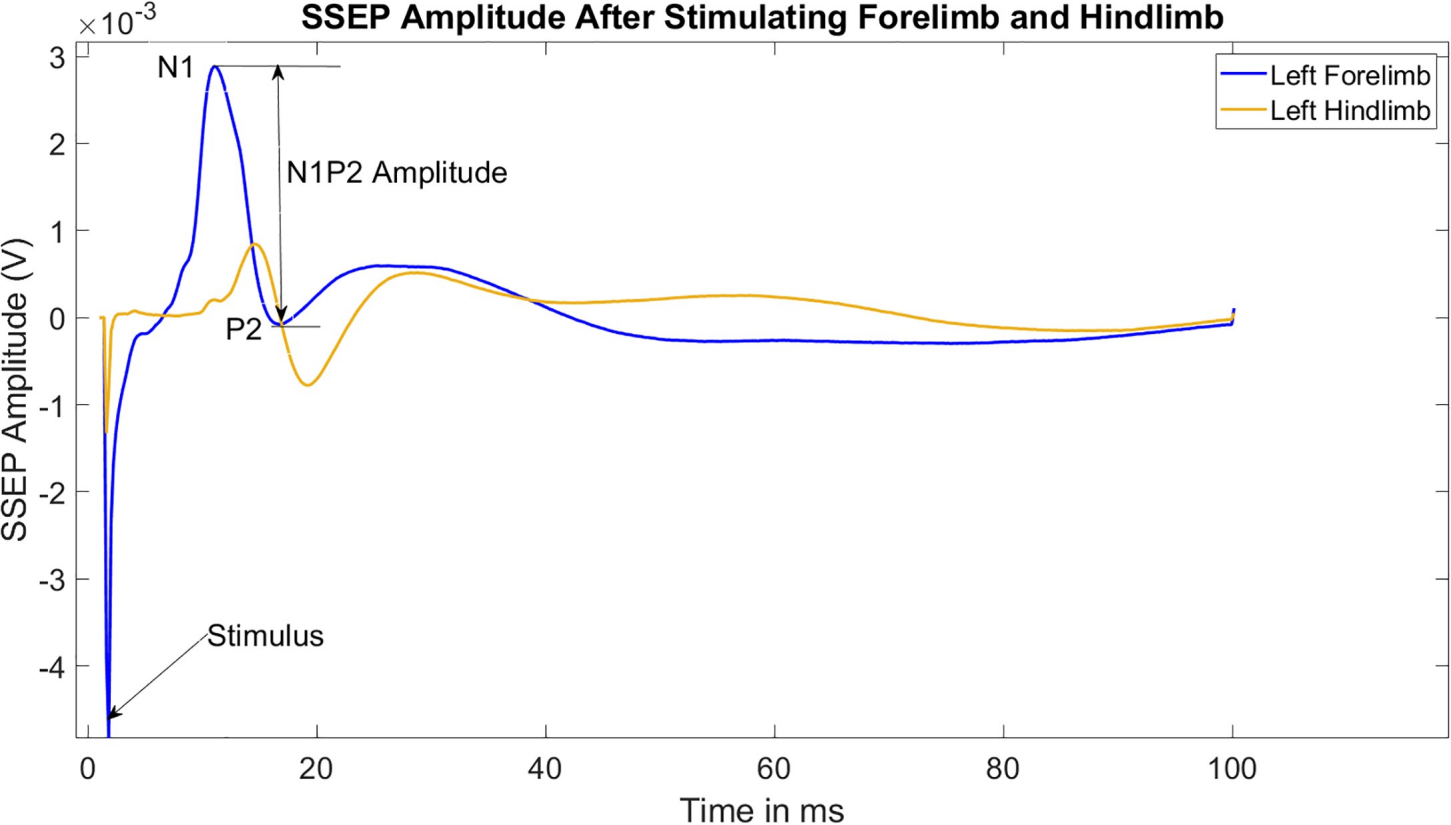
Amplitude of the averaged SSEP signals representing the baseline obtained from one subject. The stimulation sites are the left forelimb and hindlimb, and the recording sites are contralateral to the side of stimulation from the corresponding areas on the right cortices.

In general, the SSEP is mainly composed of four prominent peaks. The stimulation peaks at 5 milliseconds (ms), followed by two downward positive (P) and two upward negative (N) peaks alternatively: P1 (5–10 ms), N1 (10–15 ms), P2 (15–25 ms), and N2 (25–55 ms). The signals extended beyond 55 ms is part of the non-evoked EEG signal. Fig 3 represents two amplitudes of the averaged SSEP signals representing the baseline for stimulating left forelimb and hindlimb and recording from corresponding right cortices obtained from one subject.

## Results

The sham and control groups served as the quality control of the recordings and comparative analysis of hindlimbs SSEP signals, respectively. Evidently, since the SCI is mid-thoracic, the amplitudes of lower limbs SSEP signals are decreased substantially, indicating the progress of injury overtime. The data from sham group, control groups, and neuroprotective effect of hypothermia post-SCI on hindlimbs SSEP signals have been reported previously and are not presented here [55–58]. However, here we present the amplitude of upper limbs SSEP signals of the same two groups of rats that underwent mid-thoracic SCI, with and without receiving identical hypothermia treatment.

**Fig 4:**
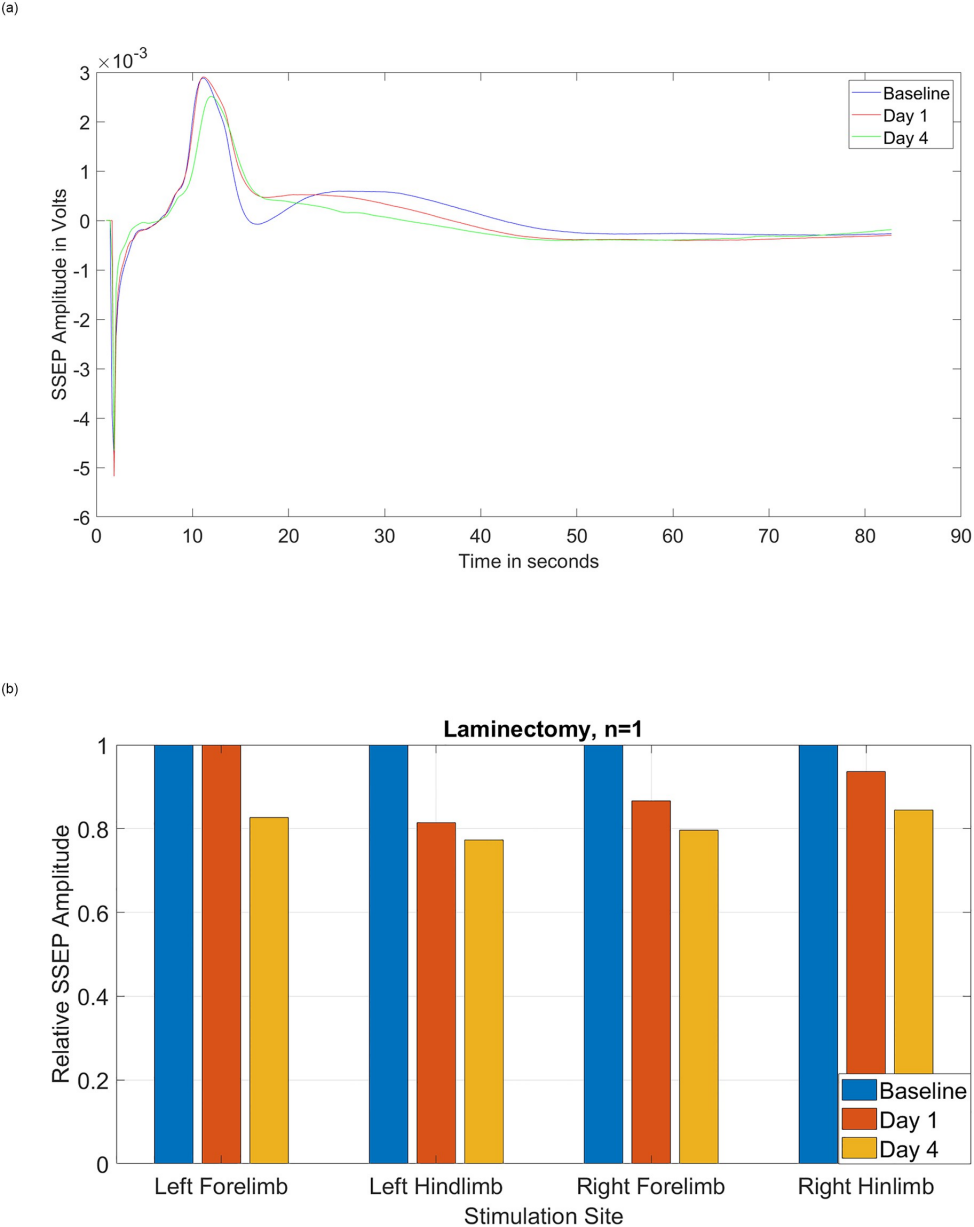
(a) Amplitude of the averaged SSEP signals obtained from one subject with laminectomy (no injury). The stimulation site is the left forelimb, and the recording site is the corresponding area of the right cortex. (b) Relative amplitude of the averaged SSEP signals obtained from one subject with laminectomy. Statistical t-test analysis with 95% confidence level demonstrates no statistically significant difference between the SSEP relative amplitudes before and after laminectomy.

The amplitude of averaged SSEP signals from three different recording days (baseline, day 1, and day 4) of one representative rat with only laminectomy (no injury and no temperature manipulation) is reported in Fig 4a. The signal obtained from left forelimb stimulation and corresponding right cortex recording is stable and shows no significant attenuation of the signals over time. This confirms that, as expected, neither inducing anesthesia, nor the laminectomy procedure has any deterioration effect on the SSEP signals. Following the same logic, Fig 4b shows the baseline, day 1, and day 4 relative amplitude of averaged SSEP signals of all four limbs of one rat in the control group with only laminectomy. As expected, because there was a stable level of anesthesia, no injury, and no temperature manipulation, the SSEP signals were consistent over time. In fact, statistical t-test analysis with 95% confidence level demonstrates that there is no statistically significant differences [left forelimb: *p = 0*.*423*, left hindlimb: *p = 0*.*187*, right forelimb: *p = 0*.*200*, right hindlimb: *p = 0*.*246*] between the SSEP relative amplitudes before (baseline), and after laminectomy on day 4 and day 7.

**Fig 5:**
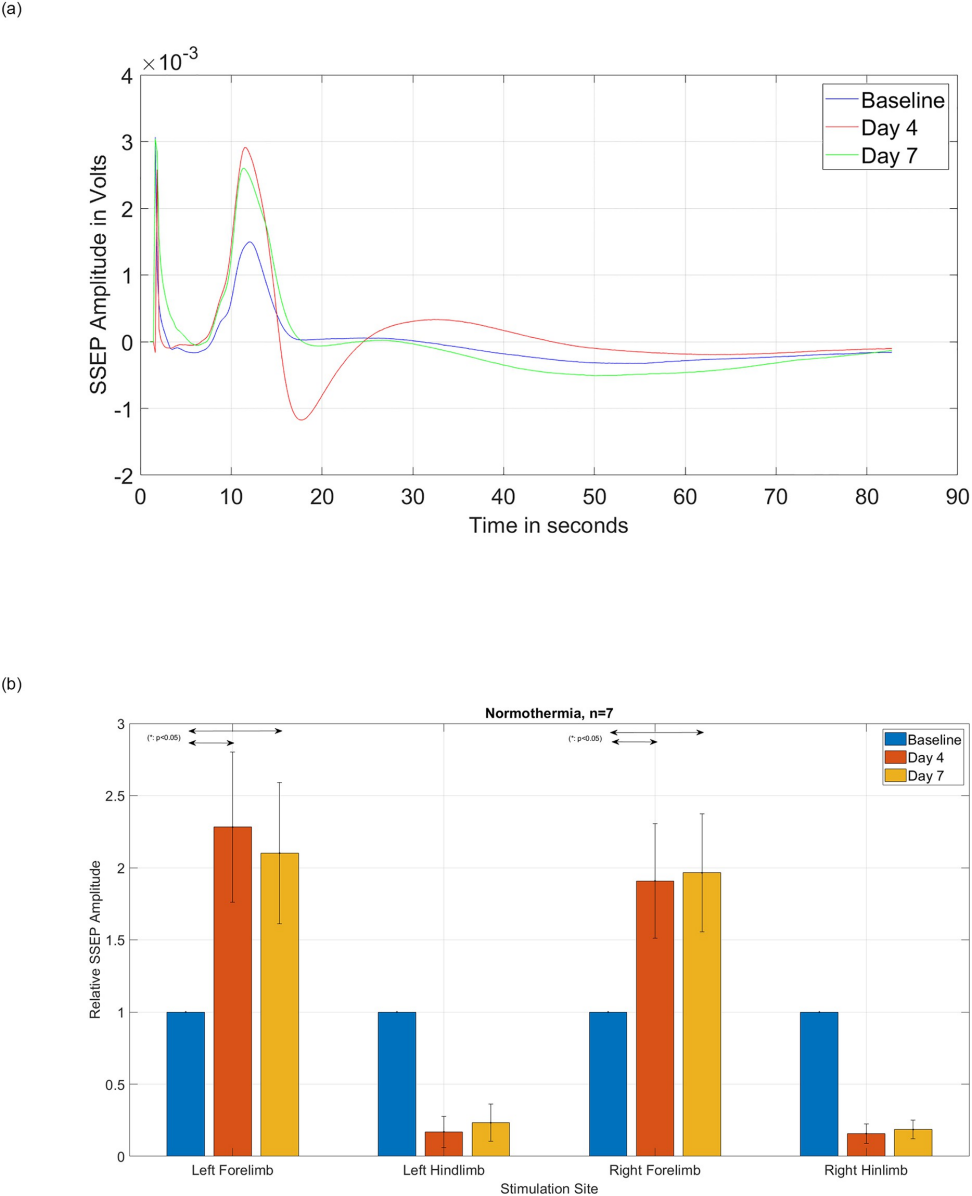
(a) Amplitude of the SSEP signals obtained after stimulating the left forelimb from one subject with moderate T8 contusive SCI, followed by normothermia. (b) Relative amplitude of the SSEP signals obtained from group (n = 7) of rats with moderate T8 contusive SCI, followed by normothermia. Statistical t-test analysis with a 95% confidence level demonstrates statistically significant differences between the SSEP relative amplitudes before and after injury in all forelimbs and hindlimbs.

Fig 5a and 5b present the SSEP signals of rats with moderate T8 contusive SCI. Rats underwent two hours of normothermia (37°C), starting two hours after SCI. The two-hour delay intends to mimic the time necessary for patients to start treatments. Fig 5a shows the amplitude of the averaged SSEP signals of one representative rat obtained from left forelimb stimulation and corresponding right cortex recording from baseline, day 4, and day 7. Fig 5b presents a significant decrease in both right and left hindlimbs SSEP amplitudes (n = 7), as expected, which represent disruption of the neuropathway at T8 post-SCI. Interestingly, it also shows the amplitudes of forelimbs SSEP signals (which are entirely above the injury site) increase soon after injury with the remarkable peak on day 4 that are extended till day 7. These changes in forelimbs SSEP amplitudes are statistically significant on both day 4 and day 7. This phenomenon has been previously described as the network neuroplasticity of upper limbs (forelimbs) neurons that compensate for the sudden dysfunction of injured lower limbs (hindlimbs) neuros. Note that the epicenter of SCI is well below the upper limb neuropathways.

**Fig 6:**
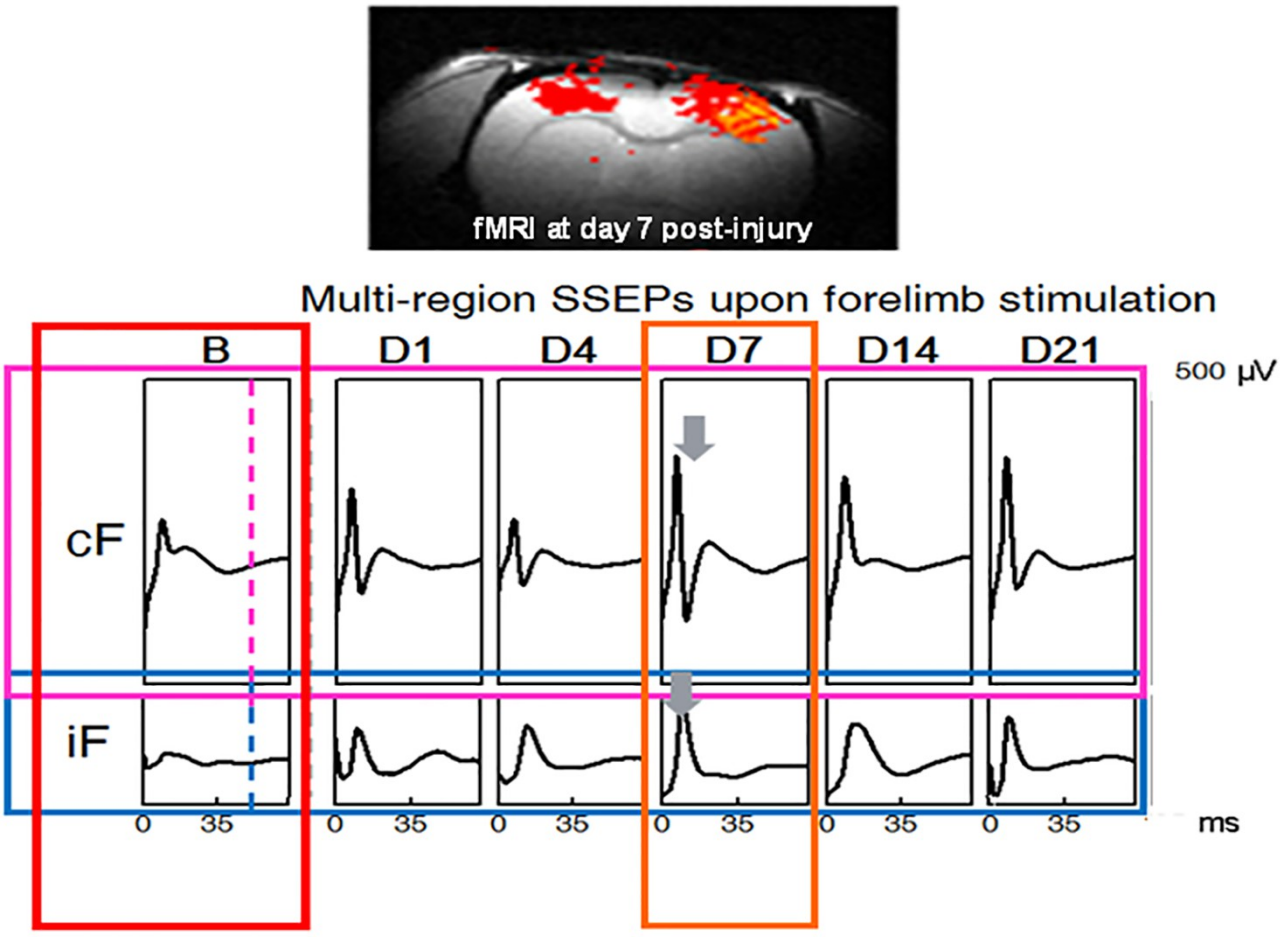
fMRI recording in rats with moderate T8 contusive SCI and normothermia. The highest changes of the SSEP amplitudes are observed on day 7 post-SCI for both contralateral and ipsilateral forelimbs cortices regions. Notably, that the SSEP amplitude changes are larger for the contralateral region. *Reproduced with permission*. *[cF*: *contralateral forelimb region*, *iF*: *ipsilateral forelimb region*, *B*: *baseline*, *& D*: *days post-SCI]*.

Fig 6 (*reproduced with permission*) shows this compensatory mechanism that is fulfilled by establishing a new reorganization of the neuronal network as well as the adoption of the higher structures and neo-plasticity of the neuronal cells in the cortices [54–58]. Statistical t-test analysis with 95% confidence level indicates that there is a statistically significant difference [left forelimb: *p = 0*.*0297* on day 4 and *p = 0*.*0442* on day 7, left hindlimb: *p = 0*.*0* on day 4 and *p = 0*.*0001* on day 7, right forelimb: *p = 0*.*0415* on day 4 and *p = 0*.*036* on day 7, right hindlimb: *p = 0*.*0* on day 4 and *p = 0*.*0* on day 7] between the means of SSEP relative amplitudes before and after injury in all limbs, in the group of rats with SCI and normothermia.

**Fig 7:**
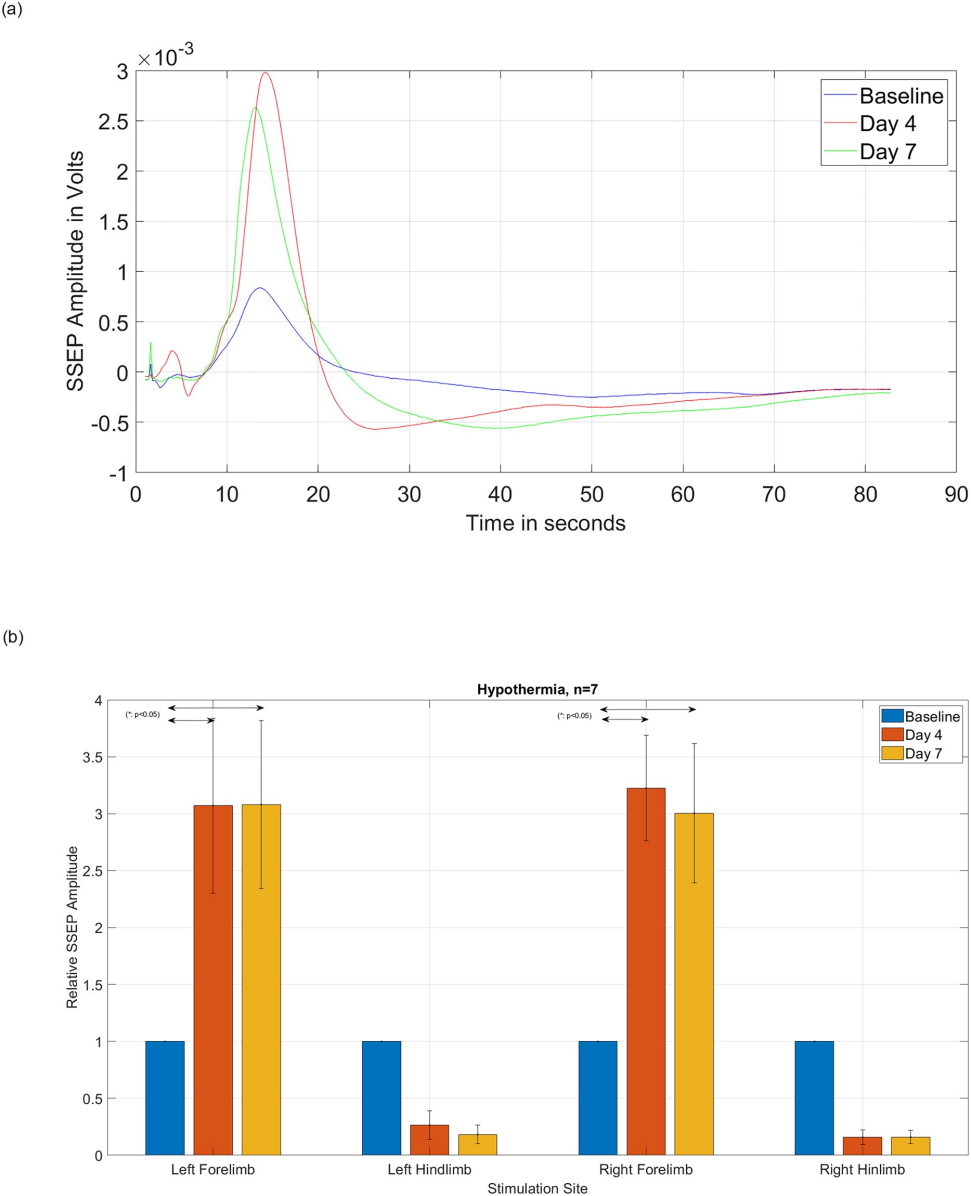
(a) Amplitude of the SSEPs obtained after stimulating the left forelimb from one subject with moderate T8 contusive SCI, followed by hypothermia. (b) Relative amplitude of the SSEP signals obtained from group (n = 7) of rats with moderate T8 contusive SCI, followed by hypothermia. Statistical t-test analysis with a 95% confidence level demonstrates statistically significant differences between the SSEP relative amplitudes before and after injury in all forelimbs and hindlimbs.

Fig 7a and 7b follow the same abovementioned reasoning with only one experimental difference. In this group (n = 7), we induced local hypothermia (32°C +/-0.5°C) in and around the T8 segment (instead of maintaining 37°C normothermia). Statistical t-test analysis with 95% confidence level demonstrates a statistically significant difference [left forelimb: *p = 0*.*0195* on day 4 and *p = 0*.*0155* on day 7, left hindlimb: *p = 0*.*0000150* on day 4 and *p = 0*.*0* on day 7, right forelimb: *p = 0*.*0004* on day 4 and *p = 0*.*0067* on day 7, right hindlimb: *p = 0*.*0* on day 4 and *p = 0*.*0* on day 7] between the means of SSEP relative amplitudes before and after injury in all limbs, in the group of rats with SCI and hypothermia. Similarly, the higher relative SSEP amplitudes of forelimbs shows that inducing early hypothermia will influence the neuroplasticity of the upper limbs network even more, when compared to the group of rats with identical injury but under the normothermia.

**Fig 8:**
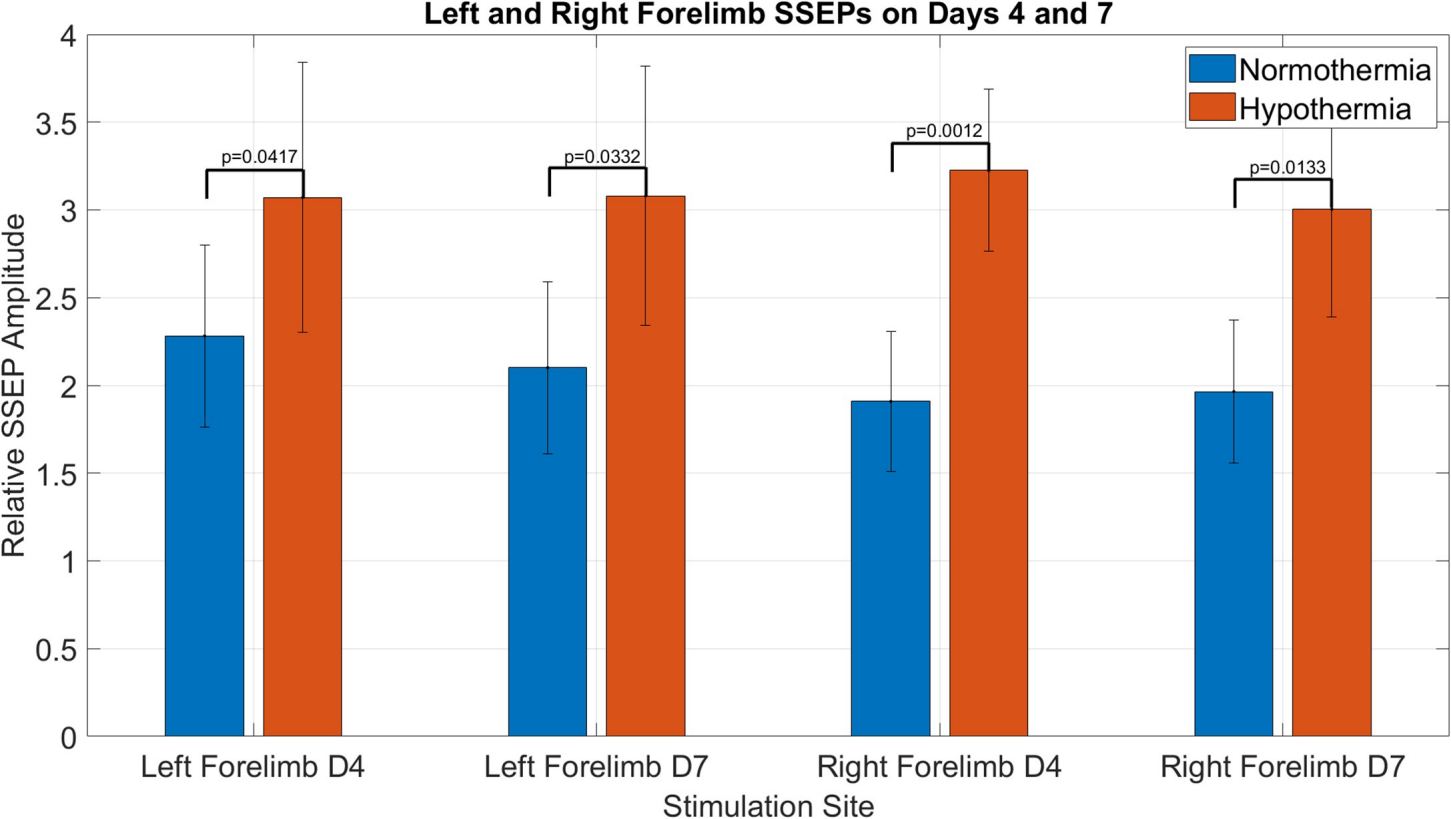
Shows the relative SSEP amplitudes obtained from stimulating left and right forelimbs of two rodent groups with moderate T8 contusive SCI, followed by normothermia in one group (n = 7) and hypothermia in the second group (n = 7). It demonstrates that there are statistically significant differences (marked areas) between the SSEP relative amplitudes with normothermia and hypothermia for both right and left forelimbs and days 4 and 7.

Fig 8 shows comparison between the relative SSEP amplitudes obtained from stimulating left and right forelimbs of two rodent groups with moderate T8 contusive SCI, followed by normothermia in one group (n = 7) and hypothermia in the second group (n = 7). As expected, there are evident changes in SSEP signals of both hindlimbs due to the injury being at T8. However, interestingly, the statistical ANOVA test applied to SSEP data from normothermia and hypothermia groups, including the baseline signals, demonstrates that there are statistically significant differences between the SSEP relative amplitudes with normothermia and hypothermia for both right and left forelimbs and days 4 and 7. The statistical ANOVA test indicates that the *p values* for statistical changes of the right forelimbs SSEP signals in injured rats with normothermia compared with the right forelimbs of injured rats with hypothermia on day 4 is *p = 0*.*0012* and on day 7 is *p = 0*.*0133*. Similarly, for the left forelimbs SSEP signals, for day 4 is *p = 0*.*0417* and for day 7 is *p = 0*.*0332*.

## Discussion and conclusion

The therapeutic benefits of inducing hypothermia for neurological disorders are controversial even today for two main reasons. One, the mechanisms of hypothermia actions are still not fully depicted, and two, the potential beneficial effects are more prominent and distinctive when early results are being evaluated and may not be much noticeable in the long term. Nevertheless, neuroscientists collectively agree that if administered correctly and in a timely manner, hypothermia can be considered a neuroprotective strategy for most neurotrauma, like traumatic brain injury and spinal cord injury. This is due to the fact that hypothermia would most probably delay the pathophysiological events post-trauma rather than prevent the progress of injury or treat them. Among various significant issues, the outcome of hypothermia treatment would depend on both the severity of the injury and the hypothermia management, which includes (i) proper timing: the sooner, the better; (ii) modality of administration: general, local, non-invasive, semi-invasive, invasive, (iii) gradation: mild, moderate, severe, (iv) duration: short-term, long-term, and (v) the rewarming protocol [4, 12, 13]. On the other hand, the beneficial effects of hypothermia for other neurological disorders are less evident. For instance, it is known that hyperthermia could be the cause or the result of epileptic seizures. Hypothermia could prevent the onset or end seizure-attacks, and this is attributed to the role of nervous system circuitries involved in thermoregulation [59].

As previously reported by others and by our team, the increase in the SSEP amplitudes of forelimbs post-mid thoracic SCI could be due to either an increased connectivity in the higher structures of the CNS between the two hemispheres (plasticity) or the new formation spinal cord network locally (re-organization of neuropathways) or both mechanisms together as a result of forelimb compensation. These findings suggest that despite the mid-thoracic location of the injury that will involve a few segments above and below the epicenter of the contusion, post-SCI plasticity can affect system-wide changes of the CNS components, including uninjured spinal cord neuropathways and brain circuits [56–58].

Figs 5 and 7 show that there are statistically significant differences due to (i) the onset of injury and (ii) the temperature manipulation in all four limbs among animal groups. Nevertheless, our main aim here was to investigate whether inducing moderate hypothermia (32°C +/-0.5°C) relatively soon (2h) after induction of contusion SCI would make any statistically significant differences in the amplitude of forelimbs SSEP signals when compared to the same but under normothermia (37°C) treatment. Our study determined that there are statistically significant differences in both forelimbs SSEP signals on day 4 and day 7 post-SCI. It is known that, unlike other organs, the central nervous system does not possess proper mechanisms for endogenous repairs or regeneration. Nevertheless, it has been demonstrated that a phenomenon, referred as plasticity and reorganization of neuropathways, would often happen after the contusive SCIs. Indeed, this phenomenon was delineated in reporting functional assessments of both hindlimbs and forelimbs in rat model of T8-contusive [1, 54, 57, 58] and T8-transection SCI [2, 55, 56]. Since the hindlimb neuropathways originate and terminate below the T8 injury site, obviously there are significant disruption within sensory and motor pathways of the lower limbs [1, 2, 34, 38]. Interestingly, on the contrary, the amplitude of forelimbs SSEP signals, which are entirely above the injury site, would increase significantly, though transiently, during the first week post-SCI. This is attributed to the neuronal network reorganization (sprouting and rewiring of surviving neurons) near the site of injury and plasticity within the corresponding neuronal cells in the cortices [54–58]. This would contribute to the endogenous compensatory mechanisms and have critical roles in determining therapeutic rehabilitation and formulating functional electrical stimulation (FES) treatment. Thus, identifying the forelimb SSEP signal variations is practical and fundamental [60–63].

In this article, we investigated the influence of early moderate semi-invasive local hypothermia induction after moderate mid-thoracic midline contusive SCI on forelimbs SSEP amplitudes and compared the results with the similar groups of rats that instead underwent normothermia. We presented the influence of early hypothermia on forelimbs neuro-electrophysiology and reported that even when the injury is mid-thoracic, the amplitude of SSEP signals of otherwise healthy forelimbs present transient but significant changes.

It is noteworthy that timely detection of plasticity and reorganization of neuropathways, as well as identification of the endogenous and exogenous factors that could influence the natural history of SCI and shape the effectiveness of any treatment, even palliative therapies, have enormous potential clinical relevance. This illustrates extensive clinical benefits in developing safe and effective rehabilitation and FES interventions and planning for long-term treatments in SCI patients.

In conclusion, statistical analysis of the relative SSEP amplitudes obtained from both forelimbs on day 4 and day 7 post-SCI of the two groups (with and without hypothermia) show remarkable functional changes of the neuropathways. Therefore, administrating moderate hypothermia soon after SCI may be considered the source of such ulterior advances of the neuro-electrophysiology signals within the neuronal networks.

## Supporting information

S1 Raw data (XLSX)
